# NH_3_-Sensing Mechanism Using Surface Acoustic Wave Sensor with AlO(OH) Film

**DOI:** 10.3390/nano9121732

**Published:** 2019-12-04

**Authors:** Xiaofeng Xu, Xiaotao Zu, Dongyi Ao, Jingxia Yu, Xia Xiang, Wanfeng Xie, Yongliang Tang, Sean Li, Yongqing Fu

**Affiliations:** 1 School of Physics, University of Electronic Science and Technology of China, Chengdu 610054, China; x.f.xu@std.uestc.edu.cn (X.X.); aodongyi@std.uestc.edu.cn (D.A.); jxyu@uestc.edu.cn (J.Y.); xiaxiang@uestc.edu.cn (X.X.); 2 Institute of Fundamental and Frontier Sciences, University of Electronic Science and Technology of China, Chengdu 610054, China; 3 School of Electronics and Information, Qingdao University, Qingdao 266071, China; wfxie@qdu.edu.cn; 4 School of Physical Science and Technology, Southwest Jiaotong University, Chengdu 610031, China; 5 School of Materials Science and Engineering, The University of New South Wales, Sydney 2052, Australia; sean.li@unsw.edu.au; 6 Faculty of Engineering and Environment, Northumbria University, Newcastle upon Tyne NE1 8ST, UK; richard.fu@northumbria.ac.uk

**Keywords:** AlO(OH) film, surface acoustic wave, ammonia sensor

## Abstract

In this study, AlO(OH) (boehmite) film was deposited onto a surface acoustic wave (SAW) resonator using a combined sol-gel and spin-coating technology, and prepared and used as a sensitive layer for a high-performance ammonia sensor. The prepared AlO(OH) film has a mesoporous structure and a good affinity to NH_3_ (ammonia gas) molecules, and thus can selectively adsorb and react with NH_3_. When exposed to ammonia gases, the SAW sensor shows an initial positive response of the frequency shift, and then a slight decrease of the frequency responses. The sensing mechanism of the NH_3_ sensor is based on the competition between mass-loading and elastic-loading effects. The sensor operated at room temperature shows a positive response of 1540 Hz to 10 ppm NH_3_, with excellent sensitivity, selectivity and stability.

## 1. Introduction

Various poisonous gases [1,2], produced daily by factories in industry and appliances used in our daily life, are hazardous toward environment and people’s health. Among poisonous gases, ammonia is one of highly toxic gases which are produced in large quantities from industry and chemical fertilizers [3,4,5]. After long-term exposure to ammonia gas, people may develop skin pigmentation or finger ulcers. In addition, ammonia can easily enter the bloodstream through alveoli, and then combine with hemoglobin to destroy the oxygen transport function. Short-term inhalation of a large amount of ammonia can cause tears, sore throat, hoarseness, cough and chest tightness [6,7,8,9]. Therefore, it is important to monitor the concentration of NH_3_ (ammonia gas) in real time in our daily life.

Various kinds of ammonia sensors have been developed in past decades, such as electrochemical sensors, metal oxide semiconductor sensors and surface acoustic wave (SAW) sensors [10,11,12,13,14,15,16]. Among these sensors, SAW sensors have advantages of excellent sensitivity, low power consumption, low operation temperature, and wireless control capabilities [17]. Semiconducting oxides such as zinc oxide (ZnO), silicon oxide (SiO_2_), titanium oxide (TiO_2_), cobalt oxide (Co_3_O_4_) and iron oxide (Fe_2_O_3_) are usually employed as sensing layers of SAW NH_3_ sensors. For example, Raj et al. reported a quartz SAW sensor with a ZnO sensitive film for ammonia sensing [18]. Wang et al. developed a SAW ammonia sensor with ZnO/SiO_2_ composite film, and its response was much higher than SAW sensor with a pristine ZnO film [19]. Tang et al. fabricated high-performance SAW NH_3_ sensors using the composite films of TiO_2_/SiO_2_, Co_3_O_4_/SiO_2_ and Fe_2_O_3_/SiO_2_ as the sensing layers, and reported that the addition of SiO_2_ could enhance the sensitivity of a SAW sensor because of its porous structures [20,21,22]. All these reported materials have porous structures and active sites for the adsorption of NH_3_. The adsorbed NH_3_ molecules interact with the composite films and cause changes of their mass, elastic modulus, or conductivity [20], which lead to the responses of the SAW sensors.

AlO(OH) (boehmite) is a positive biaxial crystal which has the characteristics of high heat resistance, good compatibility and electrical insulation, thus it can be applied effectively to lithium batterycoating materials [23,24,25,26], plastic modifiers [27], flame retardants [28]. AlO(OH) film prepared using a sol-gel method usually has a mesoporous structure and a large specific area, which is beneficial for gas sensing. In addition, it was found that there are lots of hydroxyl groups on the surface of sol-gel AlO(OH) film [29]. Hydroxyl groups have a strong affinity with water and easily form hydrogen bonds with water molecules. As a result, abundant water molecules will be adsorbed on the porous surface of the sol-gel AlO(OH) film. The adsorbed water may act as the active sites for the adsorption of NH_3_ because of the superior solubility of ammonia in water. The ammonia can react with water to form ammonia monohydrate, which may further catalyze the condensation reaction between the hydroxyl groups of AlO(OH), which can cause an increase of elastic modulus and a decrease of mass of the films. Therefore, we believe that the sol-gel AlO(OH) should be an excellent sensitive film to ammonia.

However, to the best of our knowledge, few papers have been focused on ammonia sensing using the sol-gel AlO(OH) film [30,31]. Therefore, in this paper, for the first time, we will investigate the NH_3_ sensing properties and mechanisms of a quartz SAW sensor coated with AlO(OH) sensing layer. Our results show that the film has a mesoporous structure, and abundant hydroxyl groups in the film. The mesoporous structure, hydroxyl groups and water are all found to be beneficial for the application of NH_3_ gas sensor. The sensor shows good sensitivity, selectivity and repeatability at room temperature when exposed to ammonia gas, and the sensing mechanism is identified to be influenced by the competitions between mass-loading and elastic-loading effects.

## 2. Experimental Details

**Figure 1:**
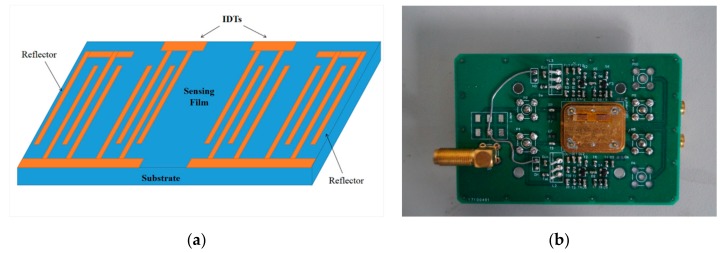
(**a**) The schematic diagram of a surface acoustic wave (SAW) resonator and (**b**) a photo of a SAW sensor.

The SAW resonator was fabricated on an ST-Cut (42°75′) quartz substrate (12 mm × 3 mm × 0.5 mm) using a standard photolithography and lift-off process with the magnetron-sputtered Al film. Figure 1a shows a schematic diagram of the SAW resonator. The resonator consists of two sets of interdigital transducers (IDTs, 30 pairs each) and reflection gratings (100 pairs). The IDTs and reflecting gratings have a periodicity of 16 μm with each finger width of 4 μm, thus the designed center frequency of the SAW resonator is ~200 MHz. The propagation direction of a SAW resonator is designed to be perpendicular to the crystallographic *x*-axis (90°-rotated), and the corresponding acoustic velocity of the SAW resonator is 3158 m/s.

For preparation of nano-alumina sol, Al(OC_3_H_7_^i^)_3_ was hydrolyzed in deionized water in a beaker for 1 h under magnetic stirring at 80 °C, and the concentration of the aluminium isopropoxide was 0.3 mol/L. Nitric acid (HNO_3_) was then added to the mixture obtained (*n*Al(OC_3_H_7_^i^)_3_:*n*HNO_3_ = 10:1) [32,33,34]. The colloidal suspension obtained was refluxed for 2 h under vigorous stirring at 80 °C. After these processes, a stable Nano-Alumina sol was obtained.

For preparation of AlO(OH) film, the nano-alumina sol was spin coated onto SAW resonator with a speed of 5000 r/min for 30 s. The coated SAW resonator was annealed in an oven at 350 °C for 1 h to obtain a stable AlO(OH) film. Finally, the annealed resonators were connected to an amplifier and phase-shift circuits to build the SAW sensor, as shown in Figure 1b.

An X-ray diffractometer (XRD, D8 ADVANCE, Bruker AXS, Karlsruhe, Germany) was used to characterize the crystallinity of the AlO(OH) films. A field-emission scanning electron microscope (FE-SEM, FEI Inspect F, Thermo Fisher, Hillsboro, OR, USA) was used to characterize the surface morphology of AlO(OH) films. A Fourier transform infrared (FTIR) Spectrometer (Nicolet 6700, Thermo Fisher, Hillsboro, OR, USA) was used to obtain the infrared absorption spectra of the prepared films. An X-ray photoelectron spectroscopy (XPS, Quantum 2000 Scanning ESCA Microprobe instrument, Physical electronics, Chanhassen, MN, USA) was used to analyze the chemical states of AlO(OH) layers. The surface area was measured based on the Brunauer–Emmett–Teller (BET) method using the instrument of ASAP-2020(Micromeritics Instrument, Atlanta, GA, USA), and the pore size distribution was derived from the adsorption branches of the isotherms using the Barrett Joyner Halenda (BJH) model.

**Figure 2:**
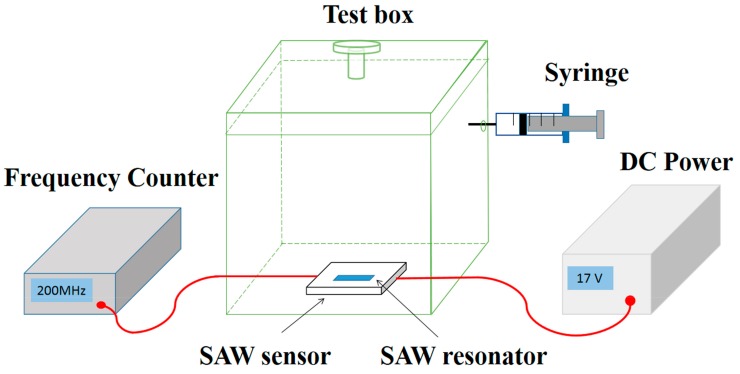
The experimental system of the gas-sensing test.

Figure 2 shows the set up of NH_3_ gas sensing system. The SAW sensor was put inside a transparent box with a volume of 20 L, and connected to a DC power (Agilent E3631A, Keysight Technologies, San Francisco, CA, USA) and a frequency counter (Agilent 53132A, Keysight Technologies, San Francisco, CA, USA). During the measurement, the temperature of the environment was maintained at 25 °C, controlled by an air conditioner. The relative humidity (RH) of the environment was adjusted using a humidifier. The testing gases (2 vol%, balanced with dry air) including C_2_H_5_OH, NO_2_, H_2_S, CO and NH_3_ were injected into the chamber using a precision syringe. For example, with 1 mL NH_3_ gas injected, the concentration of the test gas in the chamber is 1 ppm.

The response of the SAW sensor was defined as Δ*f = f*_s_ *− f*_0_, where *f*_s_ is the stable working frequency of the SAW sensor when exposed to testing gas, and *f*_0_ is the stable working frequency of the SAW sensor in the ambient environment, respectively. After the responses were recorded, the test gas was released and pure air was re-filled in the box to allow the full recovery of the sensor.

## 3. Result and Discussion

### 3.1. Structural Characterization

**Figure 3:**
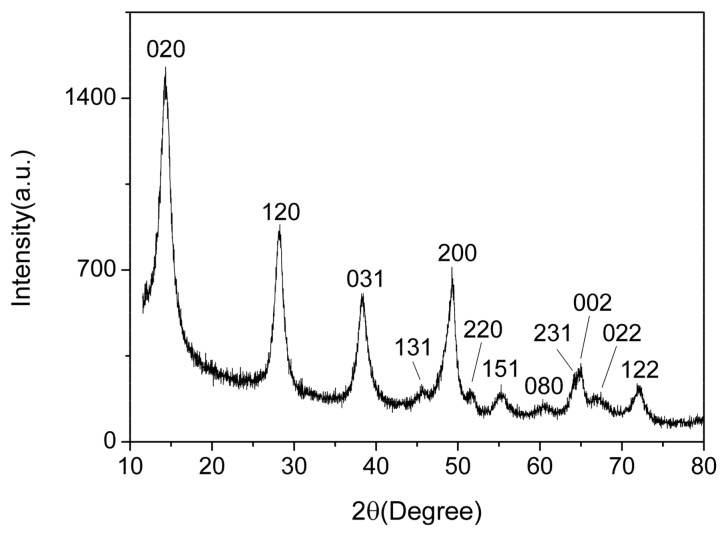
X-ray diffraction (XRD) of AlO(OH) film.

Figure 3 shows the XRD spectrum of the AlO(OH) film. There are 12 diffraction peaks located at 14.48°, 28.18°, 38.34°, 45.79°, 49.21°, 51.59°, 55.22°, 60.58°, 64.03°, 64.98°, 66.97° and 72.47°, corresponding to the crystal planes of (020), (021), (031), (131), (200), (220), (151), (080), (231), (002), (022) and (122) for AlO(OH), respectively. Compared with the standard spectrum, these 12 main diffraction peaks are consistent with the γ-AlO(OH) (JCPDS Card 21-1307). Hence, this indicates that the prepared AlO(OH) film is consisted of AlO(OH) nanoparticles. According to the Debye–Scherrer formula, we can estimate the crystallite size of the AlO(OH) nanoparticle was ~16 nm.

**Figure 4:**
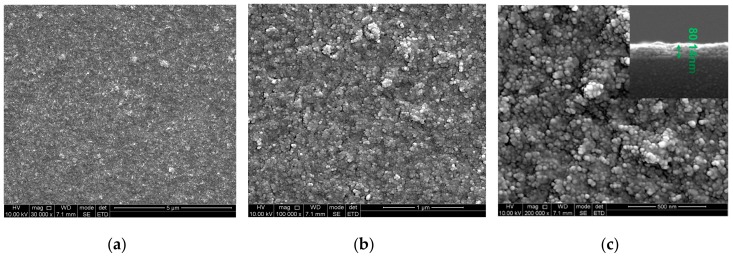
Scanning electron micrographs (SEM) of AlO(OH) film using (**a**) 30,000×; (**b**) 100,000×; (**c**) 200,000×. Inset image is the cross-sectional view of the film.

Figure 4a,b show scanning electron microscopy (SEM) images of surface morphology of the AlO(OH) film which indicates that the AlO(OH) film has a rough surface. Figure 4c shows that the film is consisted of a large amount of AlO(OH) particles with an average diameter of ~20 nm. Pores with an average diameter of tens of nanometers can be found on the film surface. The cross-sectional image of AlO(OH) film is shown in the inset of Figure 4c, which indicates the thickness of AlO(OH) film is ~80 nm.

**Figure 5:**
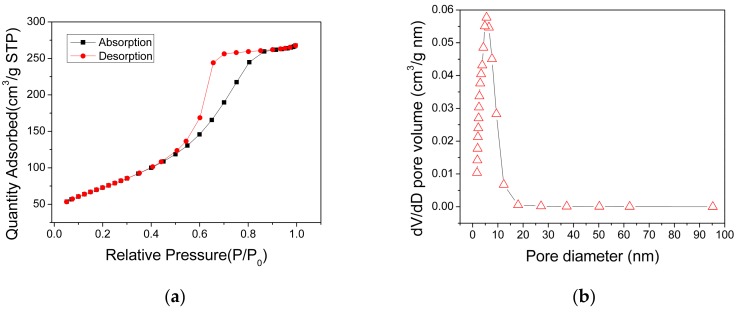
(**a**) N_2_ adsorption and desorption isotherms and (**b**) pore distribution of the AlO(OH) materials.

The specific surface areas and pore distribution of the AlO(OH) powders, which were scratched from the AlO(OH) film, were measured using the nitrogen adsorption method. The obtained N_2_ adsorption-desorption isotherm of the AlO(OH) is shown in Figure 5a, which can be categorized as type IV. It has a distinct hysteresis loop in the relative pressure range of 0.4–0.9, indicating the presence of mesopores. The measured BET surface area of AlO(OH) is 269.34 m^2^/g, and the total pore volume is 0.45 cm^3^/g. Figure 5b shows that the pore size range of the AlO(OH) material is from 2 to 9 nm and the calculated average pore diameter is 5.55 nm. These results indicate that the AlO(OH) film is mesoporous. These mesopores can provide efficient paths for gas molecules to diffuse into the film, and also provide many active surfaces for the adsorption of the gas molecules, both of which are beneficial to the gas-sensing application.

**Figure 6:**
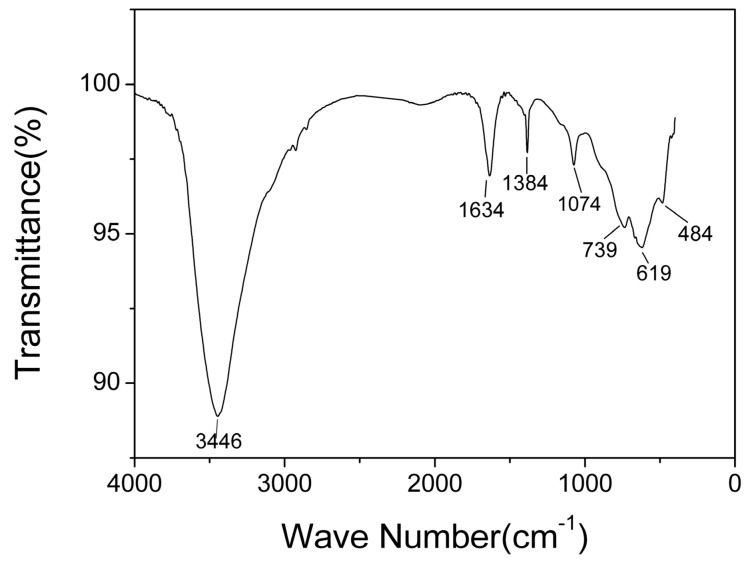
Fourier transform infrared (FTIR) spectra of AlO(OH) films.

The typical infrared spectra of AlO(OH) film are shown in Figure 6. There are obvious broad bands located around 1634 cm^−1^ and 3446 cm^−1^. The band located around 1634 cm^−1^ is caused by the bending vibration of H-O-H, which is related to free water [35]. The band around 3446 cm^−1^ corresponds to the stretching vibration of O-H, which is related to structural water and free water [35]. The bands at 1071 cm^−1^, 739 cm^−1^, and 619 cm^−1^ are generally considered to be the characteristic absorption peaks of boehmite [35]. The band at 1071 cm^−1^ corresponds to the bending vibration of Al-OH, and the bands at 739 cm^−1^, 619 cm^−1^ and 484 cm^−1^ correspond to Al-O stretching and bending vibrations [36]. The band at 1380 cm^−1^ corresponds to telescopic vibration of carboxyl C-O [37]. From the FTIR results, it can be concluded that hydroxyl groups and abundant water co-exist in the AlO(OH) film.

**Figure 7:**
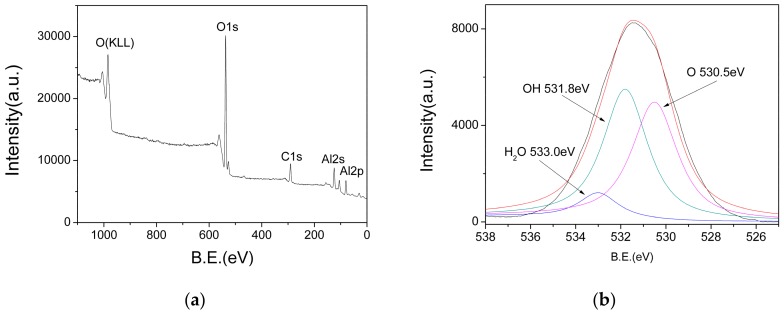
(**a**) X-ray photoelectron spectroscopy (XPS) survey spectra of AlO(OH); (**b**) Deconvoluted O1s peaks.

In order to determine the chemical composition of the AlO(OH) film, XPS measurements were performed. Figure 7a shows the XPS survey spectra, in which the peaks at 74 eV, 118 eV, 285 eV, 531 eV and 975 eV represent the binding energies of Al(2p), Al(2s), C(1s), O(1s) and O(KLL), respectively. In order to analyze the valence state of O(1s), the O(1s) peaks are deconvoluted, and the result is shown in Figure 7b. The O(1s) spectrum shows three different peaks at 533 eV, 531.8 eV and 530.5 eV, which can be assigned as those in the adsorbed water, hydroxyl and oxide ions, respectively. Therefore, these XPS results also confirm that there are abundant hydroxyl groups and water existed in the AlO(OH) film.

### 3.2. Gas-Sensing Properties

**Figure 8:**
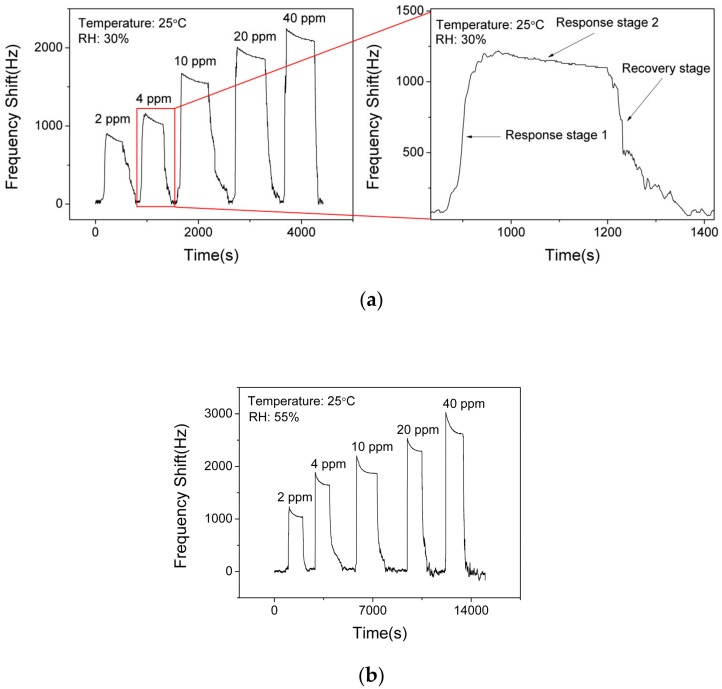
Dynamic responses and recovery of SAW sensor with AlO(OH) to various NH_3_ concentrations at the relative humidity (RH) of (**a**) 30% and (**b**) 55%.

Figure 8a shows the responses (frequency shift) of SAW sensor with the AlO(OH) layer toward NH_3_ with various concentrations of NH_3_ at a fixed RH value of 30%. The responses of the sensor are 850 Hz, 1080 Hz, 1540 Hz, 1910 Hz and 2140 Hz to a sequence of NH_3_ gas concentrations of 2 ppm, 4 ppm, 10 ppm, 20 ppm, 40 ppm, respectively. This demonstrates that the response of the sensor is increased with the increase of NH_3_ concentration. However, it becomes gradually saturated when the NH_3_ concentration is higher than 20 ppm. Based on Figure 8a, the response of the SAW sensor to ammonia can be divided into two stages. Response stage 1 shows a positive shift of the frequency response and response stage 2 shows a slight negative shift on the response, which will be discussed later.

Figure 8b presents the responses of the SAW sensor with the AlO(OH) layer toward NH_3_ with various concentrations at an RH value of 55%. The responses of the sensor are 1040 Hz, 1640 Hz, 1870 Hz, 2290 Hz and 2620 Hz to a sequence of NH_3_ gas concentrations of 2 ppm, 4 ppm, 10 ppm, 20 ppm, 40 ppm, respectively. Compared with the responses obtained at RH values of 30% and 55%, the higher value of humidity will enhance the frequency response of the sensor, and the reasons will be discussed later. This result indicates that humidity has a significant influence on a SAW sensor with AlO(OH) film.

**Figure 9:**
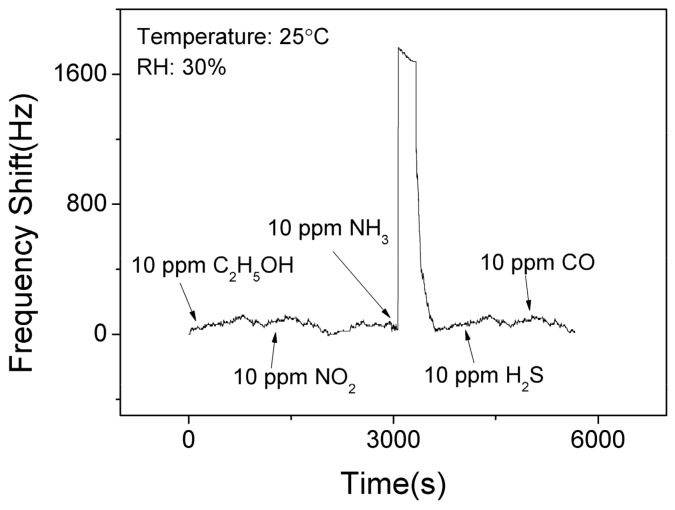
The dynamic response and recovery of the SAW sensor with AlO(OH) film to various gases.

Figure 9 shows the selectivity results of the SAW sensor with AlO(OH) film at the room temperature. It is clear that the sensor has no significant responses towards C_2_H_5_OH, NO_2_, H_2_S, and CO. However, it shows the response of 1525 Hz when exposed to 10 ppm NH_3_. This is because that there are lots of hydroxyl groups on the surface of sol-gel AlO(OH) film, and among all these gases, only the ammonia gas can further catalyze the condensation reaction between the hydroxyl groups of AlO(OH) causing an increase of elastic modulus. This test indicates that SAW ammonia sensor with AlO(OH) film has an excellent selectivity towards to NH_3_, compared with the five other gases which we have studied.

**Figure 10:**
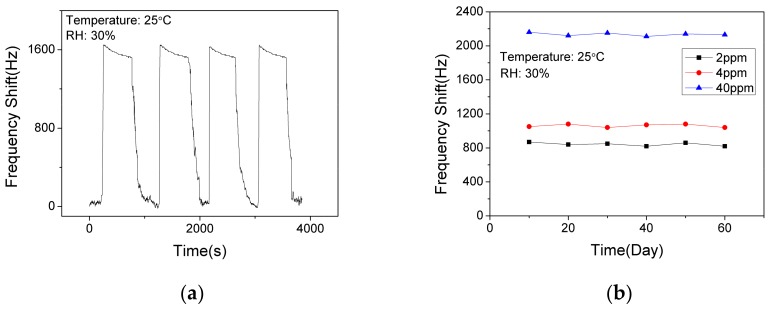
The dynamic response and recovery of the SAW sensor with AlO(OH) film (**a**) to 10 ppm NH_3_ for 4 cycles; (**b**) to NH_3_ of various concentrations in 60 days.

In order to verify the short-term stability of the SAW sensor with AlO(OH) film, the sensor was exposed to NH_3_ of 10 ppm for response and recovery for 4 cycles. As shown in Figure 10a, the response fluctuation was less than 3%, which indicates that the sensor has an excellent short-term stability. The long-term stability of the sensor was further investigated by conducting the sensing test every 10 days within a 60-day period. As shown in Figure 10b, the sensor shows similar responses to 2 ppm, 4 ppm and 40 ppm NH_3_, respectively. Thus, we can confirm that the sensor has a good long-term stability at low concentrations and high concentrations of ammonia.

The presented SAW ammonia sensors are listed in Table 1. Ma et al. exhibited a SAW sensor based on ZnO nanorod array for ammonia detection which had a negative response of 300 Hz to 50 ppm NH_3_ [38]. Wang et al. showed a SAW ammonia sensor with ZnO/SiO_2_ composite film, and its response was 1132 Hz to 10 ppm NH_3_ [19]. The SAW ammonia sensor with AlO(OH) film had a positive response of 1540 Hz to 10 ppm NH_3_. Therefore, it is meaningful to study the mechanism of the SAW ammonia sensor with AlO(OH) film.

### 3.3. Sensing Mechanisms

It has been established in literature that three effects can contribute to the SAW sensor’s responses, i.e., the changes of mass (mass loading effect), elastic modulus (elastic loading effect) and electrical conductivity (electric loading effect) [39,40,41]. Taking into account that the AlO(OH) has the characteristic of electrical insulation, the electric conductivity has little effect on the response of the SAW sensor with the AlO(OH) film [18,19]. Therefore, the response is mainly caused by elastic loading and mass-loading effects. The relationship between response and the change of elastic modulus is given by the following formula [19]:

(1) Δf=pΔE

where *p* is a positive constant, and Δ*E* is the elastic modulus change of sensing film when the SAW sensor exposed to ammonia.

The influence of change of mass on the response of the sensor follows Equation (2) [20]:

(2) $$\Delta f=(k_{1}+k_{2})\times f_{0}^{2}\times \Delta \rho_{s}$$

where *k*_1_ = −8.7 × 10^−8^ m^2^ skg^−1^ and *k*_2_ = −3.9 × 10^−8^ m^2^ skg^−1^ are the substrate material constants of ST cut quartz; *f*_0_ = 200 Mhz is the stable working frequency of the SAW sensor in the ambient environment, Δ*ρ_s_* is the area density change of sensing film when the AlO(OH) film is exposed to NH_3_. According to Equations (1) and (2), an increase of the elastic moduli and mass of the sensitive film would lead to a positive and negative response of the frequency, respectively.

**Figure 11:**
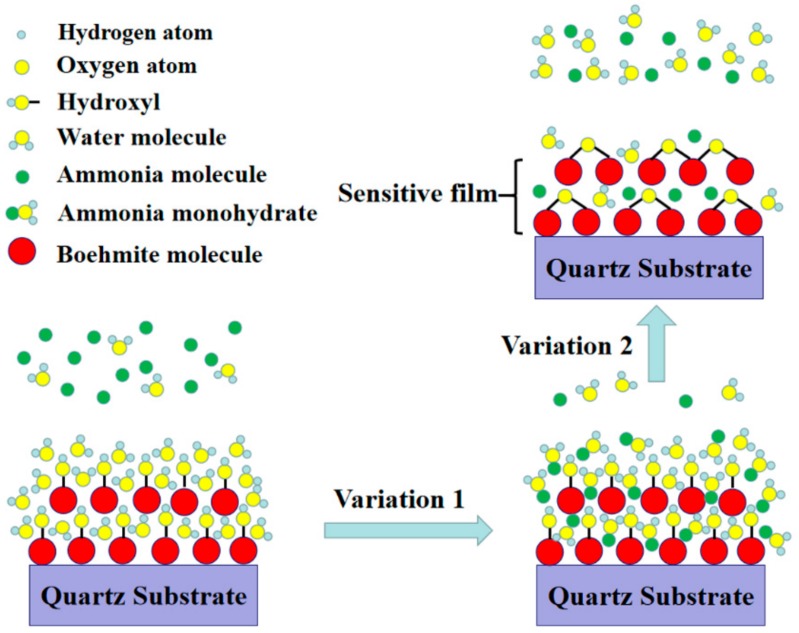
Sensing principle of AlO(OH) film with hydroxyl groups.

FTIR and XPS results have revealed that there are a large amount of hydrophilic hydroxyl groups and water on the surface of AlO(OH) films. When exposed to NH_3_ gas, the film will experience two different changes in its properties as shown in Figure 11. (1): the AlO(OH)-sensitive film has effectively absorbed the NH_3_ molecules because of the strong affinity of NH_3_ with H_2_O. The adsorbed NH_3_ will lead to an increase of the mass of the film by filling the mesopores in the film; (2): the adsorbed NH_3_ can enhance the condensation reaction between the hydroxyl groups in the AlO(OH) film, which will lead to an increase of the elastic modulus. According to Equations (1) and (2), film changes of (1) and (2) will result in either negative or positive changes of the sensor responses, respectively.

As shown in Figure 8a, when the sensor is exposed to NH_3_ gas, it initially shows a positive response (response stage 1) and then a slight decrease on the response (response stage 2). With this result, it can be concluded that both the film changes (1) and (2) contribute to the responses of the frequency. The change (2) appears to the dominant one. Therefore, the ammonia-sensing mechanism of the SAW sensor with AlO(OH) film is based on the competition of mass and elastic-loading effects.

**Figure 12:**
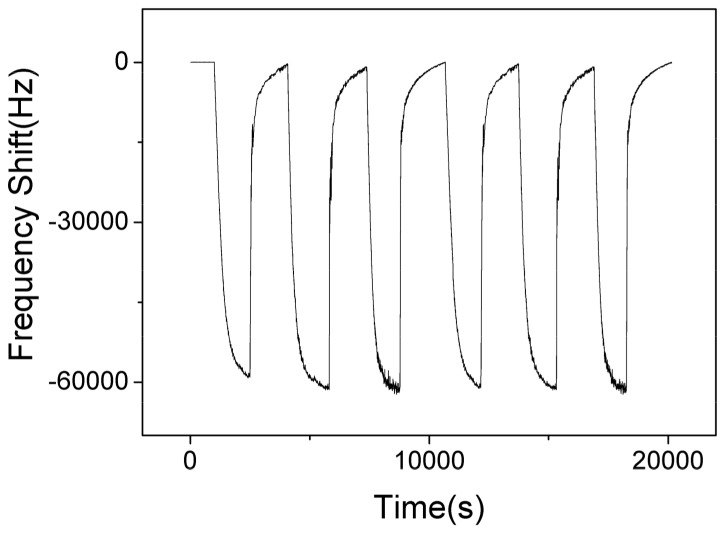
The dynamic response and recovery of SAW sensor with AlO(OH) film towards relative humidity change from 40% to 70% for 6 cycles.

Figure 12 shows the responses of SAW sensor with AlO(OH) film with the humidity level in the chamber cycled between 40% to 70% for 6 cycles. The maximum response change of the sensor is ~–60 KHz when the RH is changed from 40% to 70%. The negative response indicates that more water is adsorbed on the film at a higher humidity level, which causes an increase of mass loading on the sensor. At higher humidity, the AlO(OH) will adsorbed more water molecules, and more NH_3_ molecules can be captured by the sensitive film due to the superior affinity of NH_3_ for H_2_O, and thus the sensor may show a stronger response toward NH_3_. This can be verified from the results shown in Figure 8.

## 4. Conclusions

A SAW ammonia sensor with AlO(OH) film was systematically investigated and studied. The SEM and N_2_ adsorption results revealed the AlO(OH) film has a mesoporous structure, and the FTIR and XPS results indicated that there are a large amount of hydrophilic hydroxyl groups and water on the surface of AlO(OH) films. The mesoporous structure, hydroxyl groups, and water are all found to be beneficial for the enhanced sensitivity of the NH_3_ gas sensor. The sensor exhibits excellent stability, sensitivity and selectivity, and its ammonia-sensing mechanism is based on the competition of mass and elastic-loading effects.

## Figures and Tables

**Table 1:** The responses of ammonia SAW sensor with various films.

Film	ZnO	ZnO/SiO_2_	AlO(OH)
Response	300 Hz (50 ppm NH_3_)	1132 Hz (10 ppm NH_3_)	1540 Hz (10 ppm NH_3_)
